# Environmental enrichment does not impact on tumor growth in mice

**DOI:** 10.12688/f1000research.2-140.v1

**Published:** 2013-06-12

**Authors:** Jennifer A Westwood, Phillip K Darcy, Michael H Kershaw

**Affiliations:** 1 Sir Peter MacCallum, Department of Oncology, University of Melbourne, Parkville, 3010, Australia; 2 Department of Immunology, Monash University, Prahran, 3181, Australia

## Abstract

The effect of environmental enrichment (EE) on a variety of physiologic and disease processes has been studied in laboratory mice. During EE, a large group of mice are housed in larger cages than the standard cage and are given toys and equipment, enabling more social contact, and providing a greater surface area per mouse, and a more stimulating environment. Studies have been performed into the effect of EE on neurogenesis, brain injury, cognitive capacity, memory, learning, neuronal pathways, diseases such as Alzheimer’s, anxiety, social defeat, emotionality, depression, drug addiction, alopecia, and stereotypies. In the cancer field, three papers have reported effects on mice injected with tumors and housed in enriched environments compared with those housed in standard conditions. One paper reported a significant decrease in tumor growth in mice in EE housing. We attempted to replicate this finding in our animal facility, because the implications of repeating this finding would have profound implications for how we house all our mice in our studies on cancer. We were unable to reproduce the results in the paper in which B16F10 subcutaneous tumors of mice housed in EE conditions were smaller than those of mice housed in standard conditions. The differences in results could have been due to the different growth rate of the B16F10 cultures from the different laboratories, the microbiota of the mice housed in the two animal facilities, variations in noise and handling between the two facilities, food composition, the chemical composition of the cages or the detergents used for cleaning, or a variety of other reasons. EE alone does not appear to consistently result in decreased tumor growth, but other factors would appear to be able to counteract or inhibit the effects of EE on cancer progression.

## Introduction

Environmental enrichment (EE) for mice in laboratory conditions provides enlarged cages for large groups of mice and provides objects which stimulate enhanced sensory, cognitive, social and physical activity compared with mice housed in standard conditions. The positive effects of EE on mice (reviewed in Nithianantharajah and Hannan ^1^) have been reported from numerous studies (from a PubMed search on 9 May 2013 using the phrases “environmental enrichment” and “enriched environment”): at least 150 papers have been published showing enhanced neurogenesis, cognitive capacity, memory, learning, neuronal pathways, and improvements in diseases such as Alzheimer’s, Huntington’s, amyotrophic lateral sclerosis and brain injury. Approximately 100 papers have been published showing reduction in anxiety levels, social defeat, emotionality, depression, drug addiction, alopecia, and stereotypies. In addition, approximately 12 studies have investigated the effect of EE in infectious disease, immunity, atherosclerosis, lifespan, inflammation, asthma and obesity; and about 10 studies have dealt with its effects on olfaction, hearing, photoreceptors, and sight. Only three studies in mice have been performed on the effect of EE on cancer development or treatment.

Three papers have reported on tumor growth in mice housed in enriched environments compared with those housed in standard conditions ^2– 4^. Cao *et al.* ^2^ reported significantly decreased growth in subcutaneous (s.c.) B16 (by 43%), B16F10 (by 77%) and MC38 (by 55%) tumors in mice housed in EE conditions. Nachat-Kappes *et al.* ^3^ reported significantly smaller s.c. E0771 mammary tumors up to 10 days after tumors were inoculated orthotopically in EE housed mice, but thereafter there was no statistically significant difference in tumor size. Benaroya-Milshtein *et al.* ^4^ reported no significant difference in size of untreated s.c. 38C-13 B-cell lymphoma tumors. However, all three studies have reported statistically significant differences in other parameters between EE housed mice and standard housed mice: Cao *et al.* ^2^ reported markedly lower leptin levels and upregulation of brain-derived neurotrophic factor in EE mice, Nachat-Kappes *et al.* ^3^ reported a statistically significant increase in caspase-3 levels in the tumors of EE housed mice, and Benaroya-Milshtein *et al.* ^4^ reported reduced tumor growth and significantly greater survival of EE housed mice after immunization with an idiotype-vaccine prior to tumor injection, with 44% disease-free compared with 0% in standard cages. Thus all three studies report an influence of EE on tumors.

Our primary goal was to see if we could replicate the significant difference in tumor size found in the EE group by Cao *et al.* ^2^, who injected B16F10 s.c. and found a 77% reduction in tumor mass in EE mice and that 17% of mice had no visible tumors, compared with 0% of mice in standard caging. If we could replicate these results, this finding would have important consequences for the way in which we would need to house mice to perform future experiments when testing therapies to combat cancer.

## Materials and methods

### Cell lines

The mouse (C57BL/6) B16F10 melanoma tumor cell line ^5^ (from NP Restifo, National Cancer Institute, Bethesda, USA) was maintained in complete medium consisting of DMEM (Gibco, Life Technologies, Grand Island, NY) with 10% heat-inactivated fetal calf serum (FCS; MultiSer, Thermo Trace, Melbourne) and additives (2 mM glutamine (Gibco), 100 μg/ml streptomycin (Sigma-Aldrich, St Louis, MO) and 100 U/ml penicillin (Sigma-Aldrich) in a humidified incubator at 37°C with 5% CO _2_.

### Environmental enrichment and standard housing

**Figure 1.**
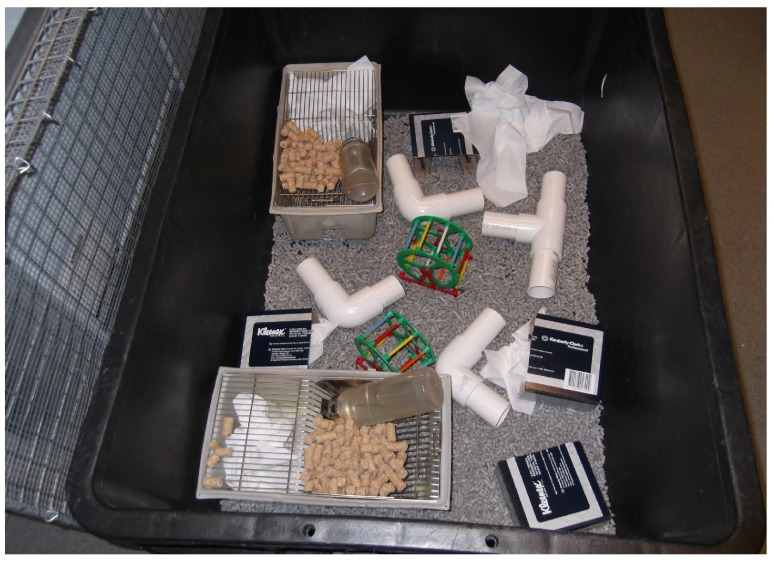
Environmental Enrichment cage setup. Setup shows the refuges, exercise wheels, and tunnels in the environmental enrichment cage.

The black low density polyethylene plastic EE cage (Plastime, Castegnero, Italy) measured 81 cm (length) × 57 cm (width) × 34 cm (height) internally, and had a wire cage lid ( Figure 1). It was stocked with the following stimulatory equipment: 2 exercise wheels, 3 PVC plumbing elbow pipes (2.54 cm diameter) bent at 90°, a 2.54 cm plumbing T-piece, 2 standard cages with holes drilled in their sides to allow mouse access and with tissues inside and pellet food provided on their wire lids, 2 cardboard boxes of tissues cut in half and inverted (refuges) and about 20 tissues scattered around. An extra EE cage was also purchased and when mice cages were cleaned fortnightly, all equipment and mice were transferred to the new cage which had clean bedding. Mice were allowed to acclimatize to their conditions for six weeks, prior to injection with tumors. The mice were not handled except for transferring during cage cleaning, and tumor measurement (on day 13 after tumor injection).

The four standard cages used measured 28 cm (length) × 14 cm (width) × 12 cm (height) internally and were made of polycarbonate plastic (Wiretainers, Melbourne, Australia).

### Specific Pathogen Free 3 (SPF3) animal facility and conditions

Fortnightly cleaning of cages was as follows: The EE cage was scraped out manually, then cage and toys were soaked for 10 mins in hot water with 2–5% Decon 90 (Decon Laboratories Ltd, East Sussex, UK) solution, scrubbed with a brush, and rinsed in hot water. The cage was left for 2 weeks before being used again, as two cages were alternated. The standard cages were scraped out manually, washed in a tunnel washer using washing machine powder, before autoclaving.

Both EE and standard cages had a layer of FibreCycle (recycled paper pellets; FibreCycle P/L, Yatala, Qld, Australia) animal bedding pellets to a depth of approximately 2 cm on the bottom of the cages, and all mice were fed with irradiated Barastoc mouse food cubes (Ridley AgriProducts, Melbourne, Australia) based on wheat, wheat byproducts, oats, meat meal, canola oil, soyabean meal, skim milk powder, molasses, salt, vitamins, and minerals. Drinking water was filtered tap water adjusted to pH 2.5–3 with hydrochloric acid.

Both EE and standard cages were housed in the same room of the animal facility. Throughout the experiment mice were maintained on a 13-hour-on : 11-hour-off lighting schedule (lights on at 6.00 am and off at 7.00 pm) in a room thermostatically maintained at 20°C. Food and water were available ad libitum. The air in the facility was not HEPA filtered or humidity controlled, and there were 15 air changes per hour.

The Specific Pathogen Free (SPF) facility houses sentinel mice in each rack and these are monitored regularly for infectious agents with the aim of detecting any pathogenic agents. The facility was monitored for the microorganisms listed in Table 1, and none of these species were detected during the period of this study. The following microbiota are detected in the mice in this facility and are considered endemic in the SPF-3 rated animal facility: Mouse Norovirus, Rotavirus, Protozoa ( *Chilomastix bettencourti* or *Entamoeba muris*, which are frequently found in intestinal tracts of normal rodents), *Proteus* spp. (probably *P. mirabilis* as this is a common inhabitant of the upper respiratory tract and faeces of normal mice), and *Helicobacter* spp.

**Table 1.** Microorganisms tested for and found absent in regular monitoring of the animal facility.

Microorganism
Mouse Hepatitis Virus
Minute Virus of Mice
Mouse Parvovirus
Theiler’s Encephalomyelitis virus
Pneumonia Virus of Mice
Sendai Virus
Murine Cytomegalovirus
Adenovirus Type 1
Reovirus Type 3
Lymphocytic Choriomeningitis Virus
Ectromelia Virus
Ectoparasites
GI Worms
*Pasteurellaceae spp.*
*Pasteurella pneumotropica*
*Streptobacillus moniliformis*
*Bordetella bronchiseptica*
*Citrobacter rodentium*
*Corynebacterium kutscheri*
*Klebsiella oxytoca*
*Klebsiella pneumoniae*
*Pseudomonas spp.*
*Salmonella spp.*
*Staphylococcus aureus*
*Streptococcus pneumoniae*
*Streptococcus spp.*
*Mycoplasma pulmonis*

### Mouse tumor model

Ethics statement: This study was carried out in strict accordance with the recommendations of the Victorian Bureau of Animal Welfare, Department of Primary Industries, and the National Health and Medical Research Council’s Australian code of practice for the care and use of animals for scientific purposes. The protocol was approved by the Peter MacCallum Cancer Centre Animal Experimentation Ethics Committee under Permit number E396. All efforts were made to minimize suffering.

Wild type male C57BL/6 mice were purchased from the Walter and Eliza Hall Institute of Medical Research (Bundoora, Australia), at age three weeks, and randomly assigned in either the EE cage (20 mice) or in four standard cages with five mice each (20 mice in this group). Mice were habituated to their cages for 6 weeks prior to tumor injection. During the habituation period two mice died (one found dead and one was culled for hydrocephalus) in the standard housed group, so that this group consisted of 18 mice for tumor injection. After the six weeks habituation, mice were shaved on the flank and inoculated s.c. with 100 μl of a single-cell suspension of 1×10 ^5^ B16F10 melanoma cells in Ca ^2+^- and Mg ^2+^-free phosphate-buffered saline (Merck, Darmstadt, Germany) (day 0). The same person injected all mice for consistency. Tumor growth was monitored using calipers, and tumor area was calculated as the product of two perpendicular diameters. Mice were culled when tumors reached 200 mm ^2^ in size or at the first signs of stress.

### Statistical analysis

Statistical significance in the experiment compared *in vivo* tumor growth and was determined by two-tailed Mann-Whitney test in Graphpad Prism (Graphpad Software, version 6.02) San Diego, California).

## Results

### Environmental enrichment did not impact on B16F10 tumor growth

C57BL/6 male mice at three weeks of age were divided into two groups. One group of 20 mice was placed in an enriched environment ( Figure 1), which consisted of a large cage (surface area of 231 cm ^2^ per mouse) with numerous pieces of stimulatory equipment (exercise wheels, tunnels, refuges, tissues), and the 18 mice in the other group were placed in four standard cages (4–5 mice/cage with surface area of 78 cm ^2^ per mouse) with tissues only.

After six weeks of habituation in their respective cages, all mice were injected subcutaneously with 1 × 10 ^5^ cells of B16F10 on their flank. Mice were not handled except for transfer to clean cages during routine fortnightly cleaning, until day 13 when all tumors were measured.

**Figure 2.**
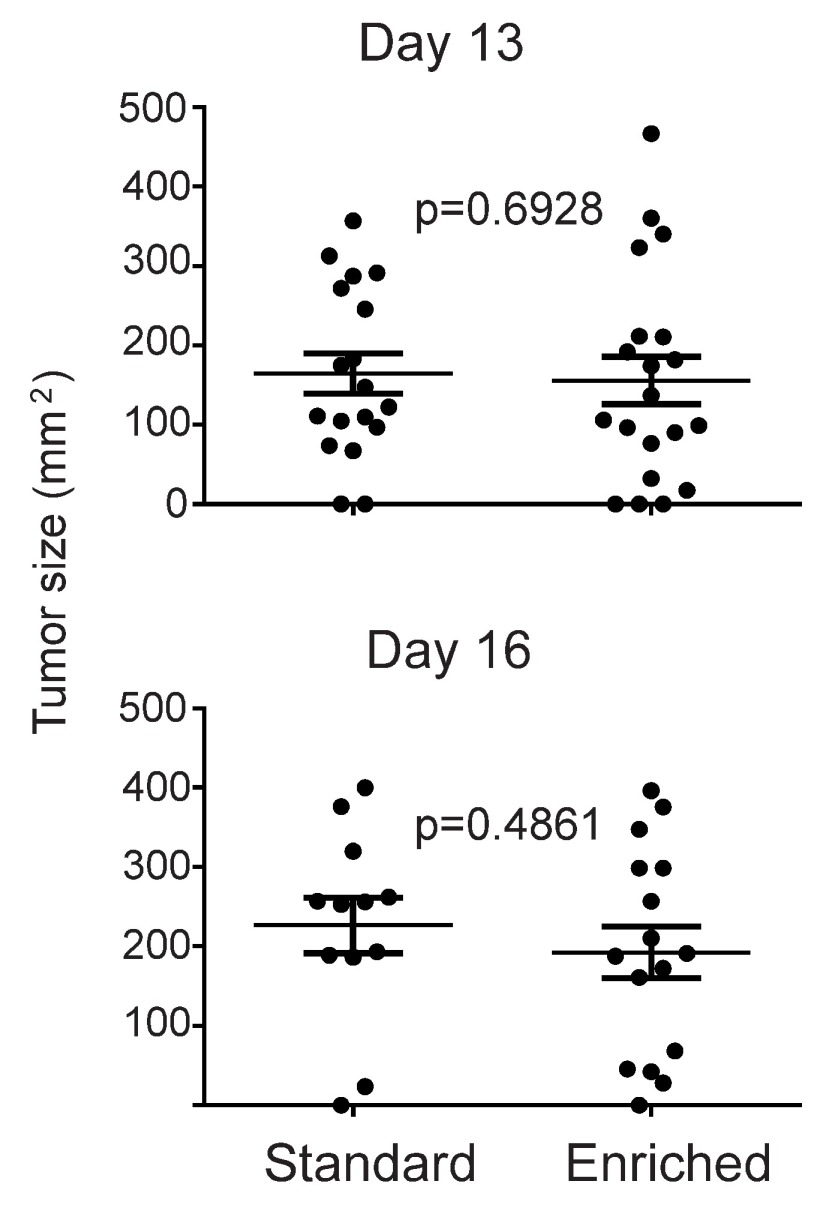
No statistical difference between B16F10 tumor size between environmental enrichment housed mice and standard housed mice. Tumor measurements shown on days 13 (first day of measurement) and 16 after tumor injection. Bar represents average measurement for the group. Error bar is ± SEM.

Figure 2 shows the tumor measurements on days 13 and 16 after tumor injection. Tumors on 33% (six of the standard group of 18 mice) and 30% (six of the EE group of 20 mice) of mice were ≥ 200 mm ^2^ on day 13 and these mice were culled on this day. The average size of tumors was 164.3 ± 25.4 (SEM) mm ^2^ (standard conditions) and 155.7 ± 29.8 (SEM) mm ^2^ (EE conditions) on day 13. Average tumor size between the two groups was not statistically significant (p=0.69). On day 16, 72% of standard housed mice (13 of the 18 mice) and 65% (13 of the 20 mice) of EE housed mice had been culled as tumors of these mice had reached the 200 mm ^2^ size threshold. All mice except one in each group had developed tumors. The experiment was terminated on day 16 as there was no significant difference between tumor sizes in the two groups.

Comparison of tumor sizes between standard housed mice and environmentally enriched housed mice Comparison of tumor sizes (mm2) between mice housed in standard and environmentally enriched cages at 13 and 16 days.

### Differences between conditions in previous studies

Table 2 summarizes the conditions for previously published studies on cancer in mice housed in EE cages, compared with the current study, in an attempt to ascertain why the results between the studies were so different. Floor space area per mouse varied from 180 to 1250 cm ^2^, with the floor space largest in the study by Cao *et al.* ^2^, which was about five times that of our study. The number of mice per EE cage varied between five and 20, but was similar between the Cao *et al.* study and our study. Enrichment toys and equipment were similar between all studies, as was the age that the mice were introduced into the cages (3–4 weeks) and the time for habituation (6–9 weeks). Tumor lines were different between studies, as were the sites of s.c. injection and the frequency of handling the mice. Mice were male in all studies except one ^3^ and of the C57BL/6 strain in all studies except one ^4^. Other parameters that could have caused variability were not mentioned in all studies, such as food composition, cage material (chemical composition and emission of fumes may vary), cage cleaning (chemical residue may vary), lighting, temperature, whether standard and EE cages were housed in the same room, bedding composition, and microbiota detected in each animal facility. With so many unspecified variables it is difficult to determine what is causing three of the studies to find no durable statistically significant difference in tumor size between EE and standard housed mice, whilst one study found a significant difference.

**Table 2.** Comparison of variables between studies studying cancer in mice housed in environmentally enriched (EE) conditions.

Variable	Benaroya-Milshtein (2007)	Cao (2010)	Nachat-Kappes (2012)	Westwood (2013)
**EE cage size (cm)**	47×30×22.5	150×150×100	60×38×20	81×57×34
**EE cage composition**	N.S.	N.S.	N.S.	Low density polyethylene
**Control cage**	N.S.	N.S.	N.S.	Polycarbonate
**EE floor space/mouse (cm ^2^)**	282	1250	180	231
**# Mice/EE cage**	5	18	10	20
**Stimulating toys/objects in** **EE cage**	2 ladders, running wheel, tunnels, 1 refuge with nesting material	2 running wheels, tunnels, igloos, 2 refuges with nesting material, huts, wood toys, a maze	1 running wheel, tunnels, igloos, cotton wool, wooden objects, 1 refuge with nesting material	2 running wheels, tunnels, tissues, 2 refuges with nesting material, cardboard huts
**Objects varied regularly?**	N.S.	N.S.	Yes	No
**Strain of mice**	C3H/eB	C57BL/6	C57BL/6	C57BL/6
**Sex of mice**	Male	Male	Female	Male
**EE, control cages in same** **room?**	N.S.	N.S.	Yes	Yes
**Lighting**	12 hour on/off	N.S.	12 hour on/off	13 hour on/11 hour off
**Temp (degrees C)**	22 ± 1	N.S.	N.S.	20
**Bedding**	Sawdust	N.S.	N.S.	FibreCycle (paper pellets)
**Humidity control?**	N.S.	N.S.	Yes	No
**Cleaning schedule**	N.S.	N.S.	N.S.	Fortnightly detailed in methods
**Food based on wheat,** **oats, meat, soy and milk?**	N.S.	N.S.	N.S.	Yes
**Microbiota endemic in** **animal facility**	N.S.	N.S.	N.S.	Norovirus, Rotavirus, Protozoa, *Proteus,* *Helicobacter*
**Age of mice put in cage** **initially**	4 weeks	3 weeks	3 weeks	3 weeks
**# weeks habituation**	6 weeks	6 weeks	9 weeks	6 weeks
**Tumor injected**	38C-13	B16F10	E0771	B16F10
**Route injected**	s.c.	s.c. on back	s.c. near mammary fat pad	s.c. on flank
**# Cells injected**	1×10 ^5^	1×10 ^5^	5×10 ^5^	1×10 ^5^
**Mouse handling** frequency*	3 times per week	day 13 and then every 2–4 days	3 times per week	day 13 and 16
**Statistical significance in** **tumor size?**	No	Yes	Not after day 10	No

* for tumor measurement; s.c., sub-cutaneous; N.S., not specified.

## Discussion

We attempted to replicate the interesting findings by Cao *et al.* ^2^, that tumors in mice housed in EE conditions grew at a significantly reduced rate, compared with mice housed in standard cages and that more EE housed mice were resistant to tumors, with 15% showing no visible tumor at day 19 (all control mice showed visible tumors).

We were not able to replicate these results, and found no statistical difference in tumor size between the two groups, even though we set up an enlarged cage with much greater floor space per mouse than in the standard cages. We also provided toys and equipment similar to Cao *et al.* to give an enriching and stimulating environment, housed a similarly large number of mice together so that there was more social interaction, introduced the mice at the same age into the cages (at 3 weeks) and habituated them for the same time (6 weeks) before tumor injection. The same tumor line (B16F10) was used, and we injected the same number of cells s.c., injected the same sex mice (male), and limited handling of the mice to the same day (day 13 post tumor injection except for cleaning).

There are several differences which may explain why we could not replicate these results. Firstly, there was a noticeable difference in growth kinetics between the B16F10 tumor line cells that we used and those used by Cao *et al.* The B16F10 tumors in our study grew faster and 30% of EE housed mice had to be culled on day 13, whereas those in the Cao *et al.* study were all still alive on day 17. In addition, the floor space per mouse was about five times greater in the Cao study than ours. Also, the toys and other objects were not identical in both studies. Problems of not standardizing EE design and lack of reproducibility of results between and within studies is reviewed by Fares *et al.* ^6^, who have attempted to remedy this by producing a standardized EE cage (for rats).

We are not claiming that EE housing cannot impact on tumor growth, but our results show that EE housing will not consistently reduce tumor growth in all animal facilities and that there may be factors which override the benefits of EE housing. These factors appear to vary between animal facilities, as other studies ^3, 4^ have also found no durable statistical difference in tumor size between the two groups.

EE housing would thus appear to offer some benefits in certain animal facilities, but these benefits may be negated or hindered in other animal facilities by other factors. These factors could consist of, for example, differences in the microbiota of the mice. Tavakkol *et al.* ^7^ examined the skin flora of mice and found 20 different species of microorganisms on the skin alone of mice in an SPF facility. There is likely to be variability in microbiota of mice in different animal facilities, and this could impact on the immune systems and limit the beneficial effect of EE housing. The impact of microbiota on the immune system, inflammation and cancer has been reviewed extensively ^8– 11^. Similarly, the food given to the mice probably varied between facilities. Diet also has an influence on microbiota ^12^. In addition, variables such as noise and number of people accessing the facility may have a negative impact on EE mice despite their enriched conditions, which may vary between animal facilities. There were many variables with no information specified in the three published studies summarized in Table 2, which could have been different in our animal facility and counteracted any benefits of EE conditions in our study. Difficulties with designing EE studies and comparison between studies to draw definitive conclusions are reviewed by Toth *et al.* ^13^, and the great variability of parameters between EE studies is reviewed in Benefiel *et al.* ^14^ and Bayne ^15^.

Our study and review of the literature has demonstrated that EE housing 20 mice in a large cage and providing toys and a stimulating environment, does not universally lead to reduced tumor growth, and that other factors appear to be acting either in concert with EE or against EE conditions to provide the variable results found.
