# Supplementary material for: Environmental enrichment does not impact on tumor growth in mice
Source: F1000Res. 2013 Jun 12;2:140. [Version 1] doi: 10.12688/f1000research.2-140.v1 (PMC3892918; doi:10.12688/f1000research.2-140.v1)
Supplement: Peer review history

# Peer review: Referee response for version 1

**Published:** 2013-06-21

**Authors:** Stephen Nutt

**Affiliations:** 1 Walter and Eliza Hall Institute of Medical Research, Parkville, USA

This is a clear study that attempts to investigate the role for environmental enrichment in the control of experimental tumour growth. Unlike the study of Cao *et al.* (2010), the authors here find that environmental enrichment does not impact on the growth of one well-characterized experimental tumour model, B16F10. The manuscript is well written and the conclusions sound. The authors thoroughly describe the similarities and differences in the environmental enrichment strategies that are employed in this and the previous studies, and provide a variety of plausible possible causes for the different findings.

Overall, this study provides a warning about the generality of any impacts of environmental enrichment on cancer cell growth that have been claimed and highlights a number of potential confounding factors. While the abstract is an appropriate summary of the study, the title is very broad and could perhaps be more restricted to encompass only the data in the manuscript, which is solely derived from one cancer cell line, B16F10 cells.

I have read this submission. I believe that I have an appropriate level of expertise to confirm that it is of an acceptable scientific standard.

# Peer review: Referee response for version 1

**Published:** 2013-06-14

**Authors:** Marc Pellegrini

**Affiliations:** 1 Walter and Eliza Hall Institute of Medical Research, Parkville, Australia

This is a nice study that adds to a growing list in the literature exploring the role of environmental enrichment on tumor pathophysiology in mice. Most of these studies, including the present, are unable to reproduce the findings of Cao *et al* in Cell 2010. The authors explore the possible explanations for these differences.

**Title and Abstract:** The abstract is a good summary.

**Article content:** Design, methods and analysis of the results are explained well and the science is robust.

Conclusions: Conclusions are sensible, balanced and justified.

**Data:** The data Is strong and presented well.

In summary, this is a very well written manuscript that attempts to dissect possible confounders in previous studies. It would have been nice if the authors could have measured serum leptin levels in their mice (to compare them with those published by Cao). If levels were similar then this would substantially undermine the conclusions of Cao. However, such measurements may be beyond the scope of the present study.

I have read this submission. I believe that I have an appropriate level of expertise to confirm that it is of an acceptable scientific standard.
